# Determinants of physical activity frequency and provider advice during pregnancy

**DOI:** 10.1186/s12884-017-1460-z

**Published:** 2017-09-05

**Authors:** Eilann C. Santo, Peter W. Forbes, Emily Oken, Mandy B. Belfort

**Affiliations:** 1 Perelman School of Medicine at the University of Pennsylvania, 3400 Civic Center Boulevard, Philadelphia, PA 19104 USA; 2 Clinical Research Program, Boston Children’s Hospital, 300 Longwood Ave, Boston, MA 02115 USA; 3 Department of Population Medicine, Harvard Pilgrim Health Care Institute, Landmark Center 401 Park Drive Suite 401 East, Boston, MA 02215 USA; 4 Department of Pediatric Newborn Medicine, Brigham and Women’s Hospital, 75 Francis Street, Boston, MA 02115 USA

**Keywords:** Physical activity, Prenatal care, Pregnancy, Barriers to exercise, Provider advice

## Abstract

**Background:**

Our aims were to (1) describe the frequency of physical activity and prenatal healthcare provider advice about physical activity during pregnancy and (2) examine determinants and correlates of 3rd trimester physical activity and receipt of physical activity advice.

**Methods:**

We analyzed data from the 2008 Pregnancy Risk Assessment Monitoring System. We studied 2669 women from North Carolina and Colorado with data on physical activity frequency in the 3 months prior to pregnancy and during the 3rd trimester and 1584 women from Oklahoma with data on provider advice regarding physical activity during pregnancy. Respondents reported physical activity, defined as 30 min or more of exercise/physical activity (excluding vocationally related activity), in in these categories: <1 day/week, 1-4 days/week, and ≥5 days/week. We defined adherence to American College of Obstetrics & Gynecology (ACOG) criteria as physical activity ≥5 days/week in the 3rd trimester. We performed logistic regression analyses weighted for sampling and adjusted for socio-demographic factors.

**Results:**

Forty-two percent of women in North Carolina and Colorado reported 3rd trimester physical activity <1 day/week, 42% 1-4 days/week, 9% ≥5 days/week; 7% reported being told not to exercise. Seventy-two percent of women in Oklahoma reported receiving physical activity advice from a prenatal care provider. Low activity frequency (<1 day/week) prior to pregnancy was strongly associated with low likelihood of ACOG guideline adherence in the 3rd trimester (aOR 0.10, 95% CU 0.04, 0.30 vs. 1–4 days/week). Underweight women were more likely to adhere to ACOG guidelines than normal weight women (aOR 2.27, 95% CI 1.36, 3.79). Overweight women were more likely to receive physical activity advice (aOR 2.9, 95% CI 1.3, 6.3 vs. normal weight), but obese women were not (aOR 0.65, 95% CI 0.4, 1.2).

**Conclusions:**

Few women meet ACOG guideline criteria for physical activity during pregnancy. Improving physical activity and weight status prior to pregnancy may improve activity levels during pregnancy. Nearly one third did not receive advice about physical activity during prenatal care. Obese women were no more likely to receive advice than their normal weight counterparts, indicating the need for targeted physical activity counseling in this population.

## Background

Regular exercise during pregnancy can improve glycemic and gestational weight control, protect against preeclampsia, and ameliorate depressive symptoms after delivery [1–3]. In tandem with diet, physical activity during pregnancy holds promise as a simple, effective intervention to improve maternal and infant health outcomes [4–6]. For this reason, the American College of Obstetrics and Gynecology (ACOG) recommends that pregnant women engage in 30 min of physical activity at least 5 days per week [7].

However, few women appear to follow these guidelines [8]. Furthermore, overweight and obese women seem to be less physically active than normal weight women [9, 10]. Specific psychological and social factors have been identified as barriers to adequate physical activity during pregnancy. Especially during the 3rd trimester, women cite fatigue, physical discomfort, and fear of harming the fetus as common reasons for low activity levels [11–14]. Interventions that focus on patient education and motivation can help women meet ACOG’s physical activity recommendations [15–20].

Some prenatal care providers may not discuss physical activity with patients. Further, some may even dissuade overweight and obese pregnancy patients from beginning exercise regimens due to misinformed safety concerns [21–23]. Given the extensive benefits of prenatal exercise for cardiovascular, mental, and metabolic health and the low rates of adherence to physical activity recommendations in pregnancy, it is important to determine the extent to which health care providers are providing appropriate advice [24]. Further identifying factors that influence whether or not a pregnant woman engages in sufficient exercise or influence whether or not she receives information regarding exercise are important to inform future interventions to improve levels of physical activity during pregnancy.

The aims of this study were 1) to describe physical activity frequency and prenatal care provider advice about physical activity during pregnancy; and 2) to identify determinants and correlates of 3rd trimester physical activity frequency and receipt of physical activity advice, specifically pre-pregnancy weight status, counseling about gestational weight gain, and sociodemographic factors.

## Methods

### Data and study cohort

We analyzed data from the 2008 (Phase 5) Pregnancy Risk Assessment Monitoring System (PRAMS). PRAMS is a national initiative of the Center for Disease Control (CDC) that collects state-specific, population-based data on maternal attitudes and behaviors regarding pregnancy. PRAMS participants were women with a recent live birth whose names were drawn from a stratified sample of eligible state birth certificate files. Each state selectively oversamples low birth weight infants and at risk socio-demographic groups to provide statistical power for smaller populations. Data collection was standardized to allow for optimal comparison in multi-state analyses. Participants were mailed an introductory pre-letter 2-4 months after delivery explaining the study prior to being sent the PRAMS questionnaire. Repeat non-responders were contacted to complete the survey by phone. Data collection is completed 60-95 days after initial mailing of the introductory pre-letter [25].

The PRAMS survey contains three sets of questions: 1) “Core” questions asked by each state; 2) “Standard” questions developed and tested by the CDC that a state may elect to include; and 3) “State-developed” questions that tailor the survey to meet specific epidemiological needs.

The Boston Children’s Hospital institutional review board determined that this study was exempt.

### Variable selection

**Fig. 1:**
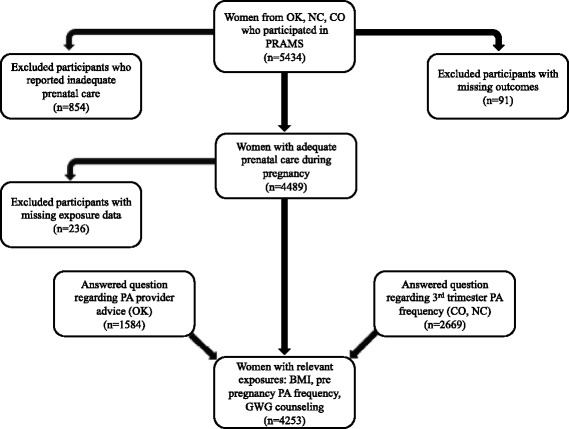
PRAMS physical activity frequency and provider advice questions

Our sample included participants from states that asked standard and state-developed questions pertaining to our main outcomes 1) physical activity frequency during the 3rd trimester (North Carolina, Colorado) and 2) receipt of provider advice about physical activity during pregnancy (Oklahoma). PRAMS defined physical activity as “physical activities or exercise for 30 minutes or more.” Frequency was categorized as: 1) Less than 1 day/week; 2) 1-4 days/week; 3) ≥ 5 days/week, or 4) Told not to exercise. Participants responded ‘yes’ or ‘no’ to an Oklahoma state developed question that asked about provider advice about exercise during pregnancy (Fig. 1).

Exposures of interest included (1) pre-pregnancy weight status (2) physical activity frequency in the 3 months prior to pregnancy, and (3) receipt of provider advice about gestational weight gain. We defined physical activity ≥5 days/week as ‘adherent to ACOG exercise recommendations’. Body mass index (BMI, kg/m^2^) was calculated by PRAMS from maternal height and weight survey data. Weight status was classified using 1990 Institute of Medicine guidelines (Committee on Nutritional Status During Pregnancy and Lactation, 1990).

**Fig. 2:**
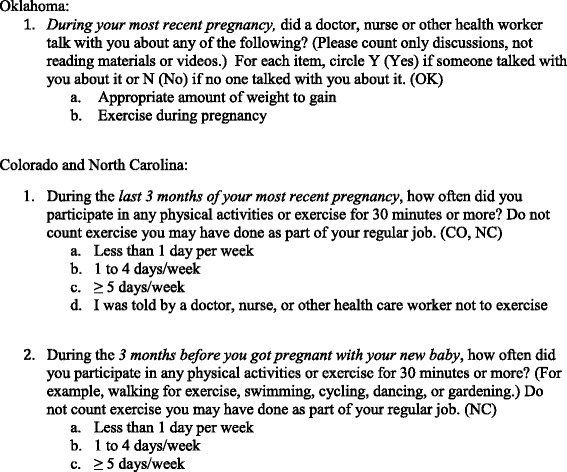
Flow of PRAMS participants included in final analysis

From our initial dataset (*n* = 5434), we excluded women who did not respond to outcome variables (*n* = 91), reported inadequate prenatal care according to the Kotelchuck index [26] (*n* = 854), or had missing exposures (*n* = 236). For bivariate analysis, we excluded women in the state of Oklahoma who reported they were ‘Told not to exercise’ by a prenatal care provider in response to query about physical activity frequency in the 3rd trimester (*n* = 281). Participant flow is shown in Fig. 2.

Compared with participants with complete data, PRAMS participants excluded from this analysis were less likely to be first time mothers (40.4 vs. 44.1%), and more likely to be unmarried (40.2 vs. 32.5%), Hispanic/ Black non-Hispanic (33.9 vs. 27.8%), very low income (<$10,000 per year) (22.1 vs. 16.7%), have fewer than 12 years (high school) of education (48.0 vs. 43.3%), be on Medicaid support (15.2 vs. 9.6%), and to be uninsured prior to pregnancy (42.8% vs. 38.2%).

We analyzed several socio-economic variables suggested by previous studies [10, 27, 28] to be correlated with frequency of physical activity before and during pregnancy. Categorical variables included: maternal education, defined by number of completed school years (0-8, 9-11, 12, 13-15, ≥16); marital status (‘married’ or ‘other’); annual household income (<$10,000, $10 -19,999, $20-34,999, $35-49,999, and ≥$50,000); maternal race/ethnicity (White non-Hispanic, African-American, Asian, Hispanic, Other); maternal age (<20, 20-34, 35-39, ≥40); and parity (0, 1, 2, ≥3). We also assessed insurance and Medicaid status before and during pregnancy. Using birth certificate information, PRAMS ranked each participant’s level of prenatal care using the Kotelchuck Index (inadequate, intermediate, adequate, adequate plus) [29].

We categorized women as ‘smokers’ (reported smoking any number of cigarettes during pregnancy), ‘non-smokers’ (reported not smoking at all), ‘drinkers’ (reported any alcohol use during pregnancy) or ‘non-drinkers’ (reported no drinking). We considered specific pregnancy complications including vaginal bleeding, premature rupture of membranes, placenta previa, symptoms of pre-term labor, hypertension, gestational diabetes, incompetent cervix, which are contraindications to aerobic exercise during pregnancy [7], and previous delivery of a low birth weight or preterm infant. Women reported these data as part of the PRAMS Core questionnaire.

### Statistical analysis

We generated weighted frequencies, percentages, and standard errors of percent, accounting for the complex survey design. We used chi-square procedures to evaluate bivariate associations of 3rd trimester physical activity frequency with pre-pregnancy weight status and physical activity frequency. Similarly, we examined associations of receiving physical activity advice with pre-pregnancy weight status and receiving gestational weight gain advice among women in Colorado and North Carolina. We further examined these associations using multivariable logistic regression. We repeated these bivariate associations after excluding participants with contraindications to 3rd trimester exercise including placenta previa, premature rupture of membranes, cerclage placement, medical bed rest, and bleeding [7]; or a history of previously delivering a low birth weight or preterm infant. Adjusting for specific socio-demographic variables, we calculated adjusted odds ratios for 3rd trimester physical activity frequency and receipt of provider advice regarding physical activity by each exposure. We used SAS version 9.3 and accounted for the complex survey design in all analyses.

## Results

Demographic characteristics, weight status, and pregnancy characteristics are shown in Table 1. A majority of the study population was white and non-Hispanic. Most had an annual household income <$50,000 and had completed education beyond high school. Over one third of the population was either overweight or obese prior to pregnancy. Common pregnancy complications included preterm labor (26% in OK, 19% in CO and NC), vaginal bleeding (14% in OK, 16% in CO and NC), pregnancy induced hypertension (16% in OK, 12.72% in CO and NC), and gestational diabetes (9% in OK, CO, and NC). During the 3rd trimester, many (41.6%) women in CO and NC reported physical activity <1 day/week, while few (9.3%) reported physical activity levels in line with current ACOG guidelines [7]. Most women in OK (72.4%) reported talking to their health care providers about physical activity during pregnancy. A slightly higher proportion (78.8%) reported receiving advice regarding gestational weight gain [Table 2].

**Table 1:** Demographic and pregnancy characteristics of PRAMS ^a^ participants from 3 states (*n* = 4253)

Variable Name	Oklahoma (*n* = 1584)		Colorado and North Carolina (*n* = 2669)	
	Weighted %	Standard Error	Weighted %	Standard Error
Maternal race				
White Non-Hispanic	68.17	2.00	66.54	1.23
Black Non-Hispanic	8.77	1.22	14.45	0.94
Asian/ Pacific Islander	0.59	0.30	2.97	0.43
Hispanic	10.98	1.39	15.23	0.90
Other	11.50	1.36	0.81	0.25
Maternal age (years)				
< 20	12.52	1.46	9.17	0.77
20-34	78.74	1.74	75.06	1.10
35-40	6.78	0.99	13.34	0.83
> 40	1.96	0.56	2.43	0.37
Maternal education (years)				
0-8	3.05	0.77	3.00	0.48
9-11	13.22	1.48	12.68	0.91
12	38.20	2.09	24.64	1.14
13-15	19.77	1.63	24.77	1.07
≥ 16	25.75	1.76	34.90	1.18
Annual household income				
< $10,000	20.31	1.80	15.70	0.99
$10-19,999	15.79	1.61	13.85	0.95
$20-34,999	24.74	1.88	16.89	1.00
$35- 49,999	11.32	1.37	11.31	0.82
> $50,000	27.85	1.87	4226	1.26
Insurance prior to pregnancy				
Yes	51.64	2.11	64.59	1.24
Medicaid status				
Yes	8.62	1.20	9.83	0.80
Smoking during pregnancy				
Yes	14.52	1.51	11.07	0.83
Drinking during pregnancy				
Yes	6.04	1.01	9.60	0.75
Adequacy of prenatal care				
Intermediate	13.41	1.46	15.32	0.88
Adequate	57.47	2.06	45.67	1.27
Adequate Plus	29.12	1.85	39.01	1.22
Pre- pregnancy weight status (BMI in kg/m2)				
Underweight (<19.8)	12.17	1.39	11.33	0.79
Normal (19.8-26)	47.79	2.10	53.08	1.27
Overweight (>26-29)	11.20	1.31	13.90	0.89
Obese (>29)	28.84	1.90	21.70	1.07
Previous live births				
0	45.33	2.10	43.78	1.26
1	31.23	1.95	31.91	1.20
2	13.45	1.38	16.89	0.97
≥ 3	10.00	1.27	7.42	0.65
Married				
Yes	61.54	2.09	69.08	1.22
Pre-term birth				
Yes	25.51	1.78	18.65	0.95
Placenta previa				
Yes	5.48	0.91	4.80	0.52
Premature rupture of membranes				
Yes	5.56	0.82	5.64	0.48
Gestational hypertension				
Yes	16.05	1.52	12.72	0.84
Gestational diabetes				
Yes	8.95	1.19	8.58	0.73
Bleeding during pregnancy				
Yes	14.35	1.42	15.89	0.92

^a^Pregnancy Risk Assessment Monitoring System

**Table 2:** Physical activity, and prenatal care characteristics of PRAMS ^a^ participants (*n* = 4253)

Variable Name	Weighted %	Standard Error
Pre- pregnancy physical activity (CO, NC)		
< 1 day/week	39.82	1.66
1-4 days/week	47.60	1.68
≥ 5 days/week	12.58	1.09
3rd trimester physical activity (CO, NC)		
< 1 day/week	41.63	1.26
1-4 days/week	42.33	1.25
≥ 5 days/week	9.33	0.69
Told not to exercise	6.71	0.61
Received physical activity advice (OK)		
Yes	72.43	1.88
No	27.57	1.88
Received gestational weight gain advice (OK)		
Yes	78.82	1.72
No	21.18	1.72

^a^ Pregnancy Risk Assessment Monitoring System

Table 3 shows unadjusted and adjusted associations of pre-pregnancy weight status and physical activity with 3rd trimester physical activity [Table 3]. Of women who followed ACOG guidelines regarding physical activity during pregnancy, 21% were underweight and 15% were obese. In contrast, of women who reported physical activity <1 day/week, 9% were underweight and 24% were obese. Physical activity level tracked from before pregnancy to the 3rd trimester, for example, 64% of women who reported physical activity <1 day/week prior to pregnancy also reported physical activity <1 day/week during the 3rd trimester. Similarly, 67% of women who were very active prior to pregnancy followed ACOG guidelines during the 3rd trimester.

**Table 3:** Predictors of 3rd trimester physical activity (PA): adjusted odds ratios (95% CI)

	3rd trimester physical activity (days/week)					
	<1 day/week	1-4 days/week	≥5 days/week	<1 vs. 1–4 days/week		≥5 vs. 1–4 days/week
	Weighted %	Weighted %	Weighted %	*P* value ^γ^	aOR^a^ (95%)	aOR^a^ (95%)
Pre-pregnancy weight status (BMI)				*0.0001*		
Underweight (<19.8)	9.04	11.84	21.16		0.81 (0.54, 1.19)	2.27 (1.36, 3.79)
Normal (BMI 19.8-26)	51.47	55.89	49.95		Reference	Reference
Overweight (>26-29)	14.71	13.06	14.40		1.16 (0.79, 1.65)	1.43 (0.78, 2.60)
Obese (>29)	24.78	19.21	14.50		1.32 (0.96, 1.80)	1.07 (0.64, 1.81)
Pre-pregnancy physical activity				*<0.0001*		
< 1 day/week	63.59	17.74	12.40		10.79 (5.46, 21.32)	0.10 (0.04, 0.30)
1-4 days/week	32.24	69.35	20.76		Reference	Reference
≥ 5 days/week	4.17	12.91	66.84		0.05 (0.02, 0.11)	1.23 (0.65, 2.33)

Table excludes women instructed ‘not to exercise’ during 3rd trimester (*n* = 281)

^a^Adjusted for maternal age, education, race, household income, adequacy of prenatal care, marital status, parity, insurance status prior to pregnancy, smoking and drinking habits during pregnancy

^ϒ^ *P* < 0.05 Chi square comparing receipt of physical activity advice among BMI and gestational weight gain advice categories

Adjusted associations shown in Table 3 demonstrate that women who reported physical activity <1 day/week prior to pregnancy were highly likely to report physical activity <1 day/week (compared with moderately active women) during pregnancy (aOR 10.8, 95% CI 5.5, 21.3) and highly unlikely to follow ACOG guidelines (aOR 0.10, 95% CI 0.04, 0.3), independent of sociodemographic and pregnancy characteristics [Table 3]. Women who were very active prior to pregnancy were inversely less likely to report physical activity <1 day/week during the 3rd trimester (aOR 0.05, 95% CI 0.02, 0.1) but more likely to follow ACOG guidelines (aOR 1.23, 95% CI 0.7, 2.3), though the latter was not statistically significant. Overweight and obesity were not significantly associated with 3rd trimester physical activity frequency in these adjusted analyses. However, compared with normal weight women, underweight women were more likely to be very active during the 3rd trimester (aOR 2.3, 95% CI 1.4-3.8).

Associations of pre-pregnancy weight status and advice about gestational weight gain with physical activity advice are shown in Tables 5 and 4. Overweight women were more likely than normal weight women to receive advice about physical activity during pregnancy (aOR 3.1, 95% CI 1.2, 7.7), but obese women were not (aOR 0.65, 95% CI 0.4, 1.2). Receiving advice about gestational weight gain was strongly associated with receiving advice about physical activity (aOR 15.2, 95% CI 8.7, 26.6). None of these associations was attenuated by adjustment for sociodemographic and pregnancy characteristics. We calculated Spearman-rank correlation between receipt of weight gain and physical activity advice. We found a linear but only moderate strength relationship correlation between these variables (r_s_ value 0.52, *p* < 0.0001).

**Table 4:** Associations of weight gain advice and pre-pregnancy weight status with prenatal physical. activity (PA) advice

Receipt of physical activity advice				
	Yes	No	*P* value ^γ^	Yes vs. No
	Weighted %	Weighted %		aOR^a^ (95%)
Pre-pregnancy weight status				
Underweight (<19.8)	11.71	13.40	*0.01*	0.98 (0.48, 2.01)
Normal (19.8-26)	48.52	45.89		Reference
Overweight (>26-29)	13.44	5.29		*3.09 (1.24, 7.71)*
Obese (>29)	26.34	35.42		0.65 (0.37, 1.15)
Received weight gain advice				
Yes	84.26	15.74	*<0.0001*	*15.22 (8.73, 26.55)*
No	28.14	71.86		Reference

^a^Adjusted for maternal age, education, race, household income, adequacy of prenatal care, marital status, parity, insurance status prior to pregnancy, smoking and drinking habits during pregnancy

^ϒ^ *P* < 0.05 Chi square comparing receipt of physical activity advice among BMI and gestational weight gain advice categories

Table 5 shows adjusted associations of demographic and pregnancy factors with adherence to ACOG guidelines and lack of prenatal physical activity advice [Table 5]. Women who reported any smoking during pregnancy were much more likely to report adherence to ACOG guidelines than women who did not smoke during pregnancy (aOR 2.06, 95% CI 1.2, 3.6). Though not significant, women who reported any alcohol intake during pregnancy were similarly more likely to report adherence to ACOG guidelines than women who did not drink (aOR 1.17, 95% CI 0.7-2.00). Higher parity was associated with not receiving provider advice about physical activity (aOR 1.9, 95% CI 1.2, 3.1), as was smoking (aOR 2.2, 95% CI 1.3, 3.9).

**Table 5:** Predictors of adherence to ACOG guidelines about physical activity (PA) and lack of PA advice during pregnancy: adjusted odds ratios (95% CI)

	Adherence to ACOG PA guidelines during 3rd trimester		Receipt of PA advice	
			(No vs. yes)	
	aOR*(95%)	*P* value ^γ^ value (95%)	aOR^a^(95%)	*P* value ^γ^
Adequacy of prenatal care				
Intermediate	0.79 (0.47, 1.32)	0.36	1.26 (0.66, 2.43)	0.61
Adequate	0.74 (0.50, 1.11)	0.14	1.20 (0.76, 1.91)	0.59
Adequate Plus	Reference	Reference	Reference	Reference
Maternal age (years)				
< 20	0.83 (0.37, 1.89)	0.66	1.21 (0.54, 2.69)	0.64
20-29	Reference	Reference	Reference	Reference
30-35	0.60 (0.34, 1.08)	0.09	1.32 (0.61, 2.85)	0.60
> 35	0.53 (0.19, 1.47)	0.22	1.00 (0.25, 4.03)	0.94
Maternal education				
< High School Education	0.58 (0.25, 1.32)	0.19	1.49 (0.64, 3.47)	0.33
High School Diploma	0.88 (0.46, 1.67)	0.70	1.14 (0.60, 2.14)	0.64
Some College	0.78 (0.47, 1.29)	0.34	1.22 (0.66, 2.26)	0.55
College Educated	Reference	Reference	Reference	Reference
Marital status				
Other	0.94 (0.56, 1.57)	0.80	0.91 (0.53, 1.59)	0.81
Married	Reference	Reference	Reference	Reference
Race/Ethnicity				
White Non-Hispanic	Reference	Reference	Reference	Reference
Black Non-Hispanic	0.88 (0.47, 1.64)	0.68	0.56 (0.23, 1.33)	0.33
Asian	1.04 (0.31, 3.44)	0.95	7.64 (0.86, 67.99)	0.06
Hispanic	1.07 (0.56, 1.79)	0.99	0.91 (0.40, 2.05)	0.82
Other	2.13(0.33, 13.262)	0.43	0.71 (0.37, 1.35)	0.31
Parity				
0	Reference	Reference	Reference	Reference
1	0.88 (0.57, 1.36)	0.56	*1.90 (1.16, 3.12)*	*0.01*
2	0.73 (0.39, 1.37)	0.33	1.02 (0.54, 1.96)	0.71
3	0.95 (0.44, 2.06)	0.90	1.85 (0.89, 3.82)	0.09
Any smoking during pregnancy				
Yes	*2.06 (1.18-3.60)*	*0.012*	*2.24 (1.28, 3.92)*	*0.006*
No	Reference	Reference	Reference	Reference
Any drinking during pregnancy				
Yes	1.17 (0.69, 2.00)	0.56	1.41 (0.62, 3.18)	0.39
No	Reference	Reference	Reference	Reference
Annual household income				
< $10 K	1.10 (0.49, 2.47)	0.83	0.88 (0.36, 2.16)	0.74
$10-19,999 K	0.68 (0.28, 1.63)	0.38	1.48 (0.65, 3.39)	0.35
$20-34,999 K	0.91 (0.48, 1.70)	0.76	1.16 (0.60, 2.22)	0.56
$35-49,999 K	0.56 (0.28, 1.13)	0.11	0.95 (0.44, 2.03)	0.90
≥ $50 K	Reference	Reference	Reference	Reference
Insurance prior to pregnancy				
Yes	Reference	Reference	Reference	Reference
No	1.01 (0.61, 1.67)	0.98	0.91 (0.53, 1.57)	0.62

^a^Adjusted for maternal age, education, race, household income, adequacy of prenatal care, marital status, parity, insurance status prior to pregnancy, smoking and drinking habits during pregnancy

^ϒ^ *P* < 0.05

We excluded 2306 women from Colorado and North Carolina with any contraindications to physical activity in sensitivity analysis. Associations between exposure and outcomes were similar in direction and somewhat stronger in magnitude (data not shown).

## Discussion

In this multi-state analysis, we found that only a small percentage of women met ACOG recommendations for physical activity during pregnancy. Specifically, only 9% of women reported being active at least 5 days per week as recommended. Nearly half of the women in this cohort reported physical activity <1 day/week during the third trimester. The strongest predictor of 3rd trimester activity level was low physical activity frequency prior to pregnancy. The majority of Oklahoma participants reported receiving physical activity advice from their prenatal care providers, and receiving provider advice about gestational weight gain was strongly predictive of also receiving advice about physical activity. Notably, while overweight women were 3 times more likely than normal weight women to receive prenatal physical activity advice, obese women were less likely than normal weight women to receive such advice.

Our low estimate of adherence to ACOG guidelines during the 3rd trimester is within the range previously described in the literature. A small (*n* = 467), Norwegian study similarly found that 11% of pregnant women engaged in moderate intensity leisure-time physical activity >3 times a week during the 3rd trimester [11]. Another study using data from the CDC’s Behavioral Risk Factor Surveillance System found that only 15% of women met ACOG guidelines for physical activity at any time in pregnancy [30]. Higher rates of adequate activity levels were reported in the First Baby Study, with an estimate of 32% of women meeting ACOG activity guidelines. However that study examined a cohort of nulliparous, predominantly white upper middle class women [16]. In contrast, our analysis is based on a representative sample of two U.S. states, with oversampling to ensure inclusion of racial/ethnic minorities and other under-represented groups. Taken together, the existing literature suggests unacceptably low levels of physical activity during pregnancy.

We found that pre-pregnancy physical activity frequency was a key determinant of adherence to ACOG guidelines during the 3rd trimester. This association has been well described in the literature, however most prior studies have been limited by small sample size and inability to adjust for sociodemographic factors [11, 27]. While our findings were not significant, our analysis supports existing literature which has found that women with higher parity and lower levels of prenatal care, income, and maternal education, are less likely to adhere to ACOG guidelines [27, 31]. Additionally, being classified as ‘underweight’ prior to pregnancy as compared to normal weight was strongly associated with adherence to ACOG guidelines and this association held even after adjustment for additional sociodemographic factors. One possible explanation is that underweight women may be more likely to have positive body image and higher self-efficacy in regards to physical activity, thus promoting continued exercise into the 3rd trimester, a time when women are most likely to stop exercising due to fatigue and physical discomfort [14, 17, 27, 31].

However, as underweight pre-pregnancy BMI is associated with many adverse pregnancy related outcomes such as low infant birth weight, pre-term birth, and NICU stay >48 h, future studies should help to clarify whether or not vigorous exercise helps modify these effects in the underweight population [32–34]. Additionally, collecting large-scale data on maternal attitudes towards weight gain/physical activity in tandem with objective data would be useful to better understand the relationship between these variables [27, 32].

Several prior studies have examined the association between smoking and physical activity levels during pregnancy. However our finding that smoking during pregnancy is associated with higher odds of adherence to ACOG guidelines is not in keeping with prior literature which has typically found an inverse, though not statistically significant, correlation [27, 31]. Petersen et al. did found a significant association between smoking and low levels of physical activity frequency in pregnancy, however this study assessed physical activity frequency based on only 1 month of data and at various points during pregnancy [31]. Other studies examining smoking as a correlate of physical activity have similarly evaluated physical activity frequency during early-mid pregnancy, possibly accounting for the discrepancy in findings [27, 35]. As smoking is associated with lower rates of gestational weight gain, women who fear gaining weight during pregnancy may be more likely to continue smoking and to exercise frequently in the 3rd trimester [36].

While the majority (72%) of women in our sample reported receiving provider advice about physical activity, a recent study of obstetric providers by Yamamoto et al. estimated that diet and exercise counseling occurred in only 18% of preventive care visits for women of childbearing age [37]. Two major differences between our studies explain the discrepant results. First, Yamamoto defined each visit as the unit of analysis, and thus could not determine how many women received advice throughout the entirety of a pregnancy. Second, those authors studied advice during visits for both pregnant and non-pregnant women, where as we focused on advice for pregnant women only. More similar to our estimate was a small (*n* = 211) 2005 study, which found that 63% of pregnant women reported talking with their obstetrician-gynecologist about physical activity [38]. Overall it appears that despite the presence of national guidelines, at least 25% of women are not receiving physical activity counseling as a routine part of prenatal care, suggesting room for improvement [20].

While overweight women were 3 times more likely than normal weight women to receive prenatal advice about physical activity, obese women were not. Yamamoto et al. found that overweight and obese women were more than 2 times more likely than normal weight women to receive advice about nutrition/exercise during prenatal care visits, but they did not separate overweight from obese women. This difference in study design is important, as providers may counsel overweight and obese groups differently [37, 39]. In particular, providers may be hesitant to recommend exercise in previously sedentary or substantially obese women even in the absence of true medical contraindications [21, 22]. Additionally, we found that multiparous women were less likely than nulliparous women to receive advice about physical activity during pregnancy. To be optimally effective, physical activity interventions may need to take into account the specific needs of pregnant women who have other children at home, for example child care and time to exercise [25].

A major strength of our study is the use of a large data set with complex survey design, which allows for state-level estimates regarding adherence to ACOG guidelines as well as provider advice regarding physical activity and weight gain. Although prior studies in hospital-based cohorts and in populations outside the U.S. have established pre-pregnancy physical activity as an important determinant of physical activity in pregnancy, our study is unique in that we incorporated state-level data from PRAMS, a survey specifically focused on pregnancy behaviors, including physical activity [11, 27]. “Core” questions and birth certificate data included in the PRAMS dataset also provide extensive information regarding socio-demographics and health, allowing for the development of adjusted models. As data is population based, it allows for greater generalizability of our findings and the ability to provide state-level recommendations for specific populations of reproductive age women to target in prenatal counseling.

We also acknowledge several limitations. Only PRAMS state-specific questions, in North Carolina, Colorado, and Oklahoma addressed physical activity and provider advice. Thus, our results may not generalize to women in other states. We were also unable to correlate receipt of advice regarding physical activity with actual 3rd trimester activity frequency, as the relevant questions were asked in different states. Specific details such as type, total duration, and intensity of physical activity were not available due to pre-defined survey response options. Similarly, details of patient-provider discussion regarding exercise during pregnancy were not available.

As PRAMS data is collected retrospectively, though on average no later than 6 months after delivery, physical activity prior to and during the 3rd trimester may have been subject to recall bias [24]. However, as pregnancy is a momentous time period for many mothers, we would argue they would be more likely to accurately remember health related behaviors, especially if asked a short time after delivery. PRAMS relies on self-reported data, including height and weight for calculations of pre-pregnancy BMI, which may lead to some misclassification of weight status. However prior studies suggest the validity of self-reported height and weight for population-based research [38, 40, 41]. Ideally, future investigations would include prospective analysis of physical activity frequency prior to and during pregnancy and incorporate both subjective and objective measures to allow for calculation of validity [39]. Additionally, because the PRAMS survey is collected in an ongoing basis, future analyses could extend our results by incorporating more recent data.

## Conclusions

In summary, we found that few pregnant women follow national guidelines regarding physical activity. High pre-pregnancy activity levels and low pre-pregnancy weight status are important determinants of adherence to ACOG exercise guidelines in the 3rd trimester. Although a majority of pregnant women report receiving physical activity advice in the context of prenatal care, at least 25% appear not to be receiving such advice, particularly multiparous and obese women. Our findings suggest that strategies to encourage weight loss and promote regular exercise prior to pregnancy, especially among women with obese or overweight BMI, may help improve levels of physical activity during pregnancy. Though to better understand determinants of pregnancy related health behaviors, increased emphasis should be placed on understanding psychological maternal factors in regards to exercise. Prenatal care providers should focus on providing appropriate advice about physical activity during pregnancy to all patients in the greater context of an individual’s psychosocial environment.

## Abbreviations

ACOG: American College of Obstetrics & Gynecology
BMI: Body mass index
PRAMS: Pregnancy Risk Assessment Monitoring System

## References

[1] Bao W, Tobias DK, Bowers K, Chavarro J, Vaag A, Grunnet LG (2014). Physical activity and sedentary behaviors associated with risk of progression from gestational diabetes mellitus to type 2 diabetes mellitus: a prospective cohort study. JAMA Intern Med.

[2] Daley AJ, Foster L, Long G, Palmer C, Robinson O, Walmsley H (2015). The effectiveness of exercise for the prevention and treatment of antenatal depression: systematic review with meta-analysis. BJOG.

[3] Mudd LME, Kelly R (2015). Review of impacts of physical activity on maternal metabolic health during pregnancy. Curr Diab Rep.

[4] Artal R, Catanzaro R, Gavard J, Mostello D, Friganza J (2007). A lifestyle intervention of weight- gain restriction: diet and exercise in obese women with gestational diabetes mellitus. Appl Physiol Nutr Metab.

[5] Rauh K, Gabriel E, Kerschbaum E, Schuster T, Von Kries R, Amann-Gassner U (2013). Safety and efficacy of a lifestyle intervention for pregnant women to prevent excessive maternal weight gain: a cluster-randomized controlled trial. BMC Pregnancy Childbirth.

[6] Sui Z, Grivell R, Dodd J (2011). Antenatal exercise to improve outcomes in overweight or obese women: a systematic review. Acta Obstet Gynecol.

[7] Gynecologists ACoOa (2002). ACOG Committee opinion. Number 267, January 2002: exercise during pregnancy and the postpartum period. Obstet Gynecol.

[8] Nascimento S, Surita F, Cecatti J (2012). Physical exercise during pregnancy:a systematic review. Curr Opin Obstet Gynecol.

[9] Renault K, Norgaard K, Andreasen K, Secher N, Nilas L (2010). Physical activity during pregnancy in obese and normal-weight women as assessed by pedometer. Acta Obstet Gynecol Scand.

[10] Fell D, Joseph K, Armson B, Dodds L (2009). The impact of pregnancy on physical activity level. Matern Child Health J.

[11] Haakstad L, Voldner N, Henriksen T, Bo K (2009). Why do pregnant women stop exercising in the third trimester?. Acta Obstet Gynecol.

[12] Evenson K, Bradley C (2010). Beliefs about exercise and physical activity among pregnant women. Patient Educ Couns.

[13] Evenson KR, Ph D, Moos M, Carrier K, Siega-riz M, Ph D (2010). Perceived barriers to physical activity among pregnant women. Matern Child Heal J.

[14] Clarke PE, Gross H (2004). Women's behaviour, beliefs and information sources about physical exercise in pregnancy. Midwifery.

[15] Hawkins M, Chasan-Taber L, Marcus B, Stanek E, Braun B, Ciccolo J (2014). Impact of an exercise intervention on physical activity during pregnancy: the behaviors affecting baby and you study. Am J Public Health.

[16] Kraschnewski JL, Chuang CH, Downs DS, Weisman CS, McCamant EL, Baptiste-Roberts K (2013). Association of Prenatal Physical Activity and Gestational Weight Gain: results from the first baby study. Womens Health Issues.

[17] Melton B, Marshall E, Bland H, Schmidt M, Guion WK (2013). American rural women's exercise self-efficacy and awareness of exercise benefits and safety during pregnancy. Nurs Health Sci.

[18] Pearce EE, Evenson KR, Downs DS, Steckler A (2013). Strategies to promote physical activity during pregnancy. Am J Lifestyle Med.

[19] Duthie EA, Drew EM, Flynn KE (2013). Patient-provider communication about gestational weight gain among nulliparous women: a qualitative study of the views of obstetricians and first-time pregnant women. BMC Pregnancy Childbirth.

[20] Basu A, Kennedy L, Tocque K, Jones S (2014). Eating for 1, healthy and active for 2; feasibility of delivering novel, compact training for midwives to build knowledge and confidence in giving nutrition, physical activity and weight management advice during pregnancy. BMC Pregnancy Childbirth.

[21] Power M, Cogswell M, Schulkin J (2006). Obesity prevention and treatment practices of U.S. obstetrician–gynecologists. Obstet Gynecol.

[22] Bauer P, Broman C, Pivarnik J (2010). Exercise and pregnancy knowledge among healthcare providers. J Women's Health.

[23] Stengel MR, Kraschnewski Jennifer L, Hwang Sandra W, Kjerulff Kristen H, Chuang CH (2012). What my doctor Didn’t tell me: examining health care provider advice to overweight and obese pregnancy women on gestational weight gain and physical activity. Womens Heal Issues.

[24] May L, Suminski R, Linklater E, Jahnke S, Glaros A (2013). Exercise during pregnancy: the role of obstetric providers. J Am Osteopath Assoc.

[25] Centers for Disease Control and Prevention. Methodology - PRAMS. http://www.cdc.gov/prams/methodology.htm. (accessed May 2017). Updated April 1, 2016. Accessed 2 Jun 2017.

[26] Kotelchuck M (1994). An evaluation of the Kessner adequacy of prenatal care index and a proposed adequacy of prenatal care utilization index. Am J Public Health.

[27] Gaston A, Cramp A (2011). Exercise during pregnancy: a review of patterns and determinants. J Sci Med Sport.

[28] Pillman M (2007). Predictors of change in physical activity during and after pregnancy: project viva. Am J Prev Med.

[29] Kotelchuck M (1994). The adequacy of prenatal care utilization index: its US distribution and association with low birthweight. Am J Public Health.

[30] Evenson KR (2004). David, Savitz a, Huston SL. Leisure-time physical activity among pregnant women in the US. Paediatr Perinat Epidemiol.

[31] Petersen A, Leet T, Brownson R (2005). Correlates of exercise among pregnant women in the United States. Med Sci Sports Exerc.

[32] Downs DS, DiNallo JM, Kirner TL (2008). Determinants of pregnancy and postpartum depression: prospective influences of depressive symptoms, body image satisfaction, and exercise behavior. Ann Behav Med.

[33] Schummers L, Hutcheon JA, Bodnar LM, Liberman E, Himes KP (2016). Risk of adverse pregnancy outcomes by Prepregnancy body mass index: a population-based study t inform pregnancy weight loss counseling. Obstet Gynecol.

[34] Gilboa SM, Correa A, Alverson C (2008). Use of a spline regression in an analysis of maternal prepregnancy body mass index and adverse birth outcomes: does it tell us more than we already know?. Ann Epidemiol.

[35] Ning Y, Williams J, Dempsey T (2003). Correlates of recreational exercise in early pregnancy. J Matern Fetal Neonatal Med.

[36] Lindberg S, Anderson C, Pillai P, Tandias A, Arndt B, Hanrahan L (2016). Prevalence and predictors of unhealthy weight gain in pregnancy. WMJ Off Publ State Med Soc Wisconsin.

[37] Yamamoto A, McCormick M, Burris H (2014). US provider-reported diet and physical activity counseling to pregnant and non-pregnant women of childbearing age during preventive care visits. Matern Child Health J.

[38] Krans EEGJ, Dubbert PM, Pm K, Miller AL, Replogle WH (2005). Pregnant women's beliefs and influences regarding exercise during pregnancy. J Miss State Med Assoc.

[39] de Jersey S, Nicholson J, Callaway L, Daniels L (2013). An observational study of nutrition and physical activity behaviours, knowledge, and advice in pregnancy. BMC Pregnancy Childbirth.

[40] Shin D, Chung H, Weatherspoon L, Song W (2014). Validity of Prepregnancy weight status estimated from self-reported height and weight. Matern Child Health J.

[41] Evenson KR, Chasan-Taber L, Downs DS, Pearce EE (2012). Review of self-reported physical activity assessments for pregnancy: summary of the evidence for validity and reliability. Paediatr Perinat Epidemiol.

